# Supplementary material for: A Systematic Review of Atypical Endometriosis-Associated Biomarkers
Source: Int J Mol Sci. 2022 Apr 17;23(8):4425. doi: 10.3390/ijms23084425 (PMC9029517; doi:10.3390/ijms23084425)
Supplement: Supplementary file 1 [file ijms-23-04425-s001.zip › ijms-1661946-supplementary.pdf]

Supplementary Materials: Supplementary Figure S1: Risk of bias assessment according to the risk of bias tool by Clarity Group [19].

Risk of bias domains

Study	D1	D2	D3	D4	D5	Overall
Chalas et al. 1990 (20)	+	+	+	-	-	+
Ogawa et al. 2000 (21)	+	+	+	-	-	+
Bayramoglu et al. 2001 (22)	+	+	-	-	-	-
Nezhat et al. 2002 (23)	+	+	-	-	-	-
Del Carmen et al. 2003 (24)	+	+	X	X	-	-
Amemiya et al. 2004 (25)	X	X	-	X	X	X
Saìnz de la Cuesta et al. 2004 (26)	+	+	+	+	+	+
Akahane et al. 2005 (27)	X	-	X	-	X	X
Kato N et al. 2006 (15)	+	+	+	-	-	+
Ali-Fehmi et al. 2006 (28)	+	+	-	-	-	-
Akahane et al. 2007 (17)	+	-	+	-	-	-
Finas et al. 2008 (29)	-	X	-	X	X	X
Yamamoto et al. 2008 (30)	+	+	-	-	-	-
Wiegand et al. 2010 (11)	-	X	X	X	X	X
Yamamoto et al. 2010 (31)	+	-	+	+	-	+
Yamamoto et al. 2011 (14)	+	-	+	+	-	+
Kato et al. 2012 (32)	+	+	-	-	+	+
Xiao et al. 2012 (33)	-	-	+	-	-	-
Yamamoto et al. 2012 (34)	+	+	+	+	+	+
Yamamoto et al. 2012 (35)	+	-	+	-	-	-
Yamamoto et al. 2012 (36)	+	-	+	+	-	-
Lai et al. 2013 (37)	+	X	X	X	X	X
Vercellini et al. 2013 (38)	+	-	+	-	-	-
Lin et al. 2014 (39)	+	+	+	+	-	+
Suryawanshi et al. 2014 (40)	+	+	+	+	-	+
Anglesio et al. 2015 (41)	+	X	X	X	X	X
Matsumoto et al. 2015 (42)	-	-	X	X	-	-
Er et al. 2016 (43)	-	X	X	X	X	X
Kato et al. 2016 (44)	+	+	+	+	-	+
Ma et al. 2016 (45)	+	+	+	-	-	+
Stamp et al. 2016 (46)	+	+	+	-	-	+
Andersen et al. 2018 (47)	+	+	+	+	+	+
Zhang et al. 2018 (48)	+	-	-	-	-	-
Niguez-Sevilla et al. 2019 (49)	+	+	+	+	+	+
Jiao et al. 2019 (50)	+	-	-	X	X	-
Păvăleanu et al. 2020 (51)	+	-	+	-	X	-
Penciu et al. 2020 (52)	+	-	X	X	X	X
Lenz et al. 2021 (53)	+	+	-	X	-	-
Shin et al. 2021 (54)	+	+	+	-	-	-

Domains:
D1: Bias arising from the randomization process.
D2: Bias due to deviations from intended intervention.
D3: Bias due to missing outcome data.
D4: Bias in measurement of the outcome.
D5: Bias in selection of the reported result.

Judgement
X High
- Some concerns
+ Low
